# Conceiving and evaluating novel therapeutic strategies with patients and peer practitioners: The case of urban remediation program

**DOI:** 10.1192/j.eurpsy.2021.1365

**Published:** 2021-08-13

**Authors:** L. Abrahamyan Empson

**Affiliations:** Department Of Psychiatry, Chuv, Hôpital de Cery, Prilly, Switzerland

**Keywords:** Psychoses, Urbanicity, recovery, Particpatory design

## Abstract

**Introduction:**

While extensive recent data details risk factors for psychoses in urban milieu, insights regarding recovery processes in cities are scarce. This hampers the translation of promising epidemiological and neuroimagery findings into effective therapeutic strategies. Given the twofold higher incidence of psychoses in cities and the fact that 68% of world population will be urban by 2050, it becomes an urgent matter of psychiatric care.

**Objectives:**

This presentation details specific targets for therapeutic interventions in city context to further discuss a pioneering participatory project with the aim to conceive a novel city specific recovery-oriented program.

**Methods:**

Based on most recent research data, some of which our own, a comprehensive survey of urbanicity studies and an overview of main avenues for developments will be presented.

**Results:**

Urban milieu is a complex dwelling space made of protective and disruptive features. During each life course they may form unique combinations hampering or enhancing psychological well-being. Urban living is not only correlated with higher prevalence of psychoses, but also with better access to health care and lower rates of treatment resistant schizophrenia, pointing to some beneficial aspects of city living on recovery processes. The interplay between personal characteristics, urban resources and supportive social environments seems pivotal to recovery calling for multilevel interventions (CBT interventions, peer-support, go-alongs, resocialization) and integration of different stakeholders (patients, peer-practitioners, community actors).

**Conclusions:**

Participatory approach (design thinking, urban lab etc.) represents an important means of innovation and ensures the best match between patients needs and therapeutic propositions.

